# Periodicity of Fruit Cracking in Orange Fruit and Integrated Management Intervention

**DOI:** 10.3390/plants14030389

**Published:** 2025-01-27

**Authors:** Xingjian Shi, Mingxia Wen, Zhihao Dong, Jiangzhou Zhang, Anoop Kumar Srivastava, Mohamed G. Moussa, Yueqiang Zhang

**Affiliations:** 1 Zhejiang Institute of Citrus Research, Taizhou 318026, China; 2 College of Resources and Environment, China Agriculture University, Beijing 100193, China; shixj20152022@163.com (X.S.); dongzh@mail.hzau.edu.cn (Z.D.); 3 Guangxi Academy of Specialty Crops, Guilin 541004, China; shixj20152022@163.com; 4 College of Resources and Environment, Inner Mongolia Agricultural University, Hohhot 010010, China; jzzhang@fafu.edu.cn; 5 Indian Council of Agricultural Research-Central Citrus Research Institute, Nagpur 440033, Maharashtra, India; aksrivas2007@gmail.com; 6 International Center for Biosaline Agriculture, Dubai P.O. Box 14660, United Arab Emirates; mohamedgomaa_ali@agr.asu.edu.eg; 7 College of Resources and Environment, Southwest University, Chongqing 610072, China; zhangyq82@swu.edu.cn

**Keywords:** citrus, cracking, nutrients, soil, yield

## Abstract

Fruit cracking in citrus is one of the most researched constraints in crop management. However, researchers are still clueless even today on how to curtail this important production loss through an integrated management system. Our study introduces a management strategy for fruit cracking in citrus by analyzing different production constraints. As many as 70 Bingtang orange (*Citrus sinensis* L. Osbeck cv. *Bingtang*) orchards in Xinping County were investigated to determine the intensity and periodicity of fruit cracking. The results indicated that citrus cracking was in a high incidence state during production in the past two years, accounting for 48.2–50.6% of fruit drop following the physiological premature drop period, particularly exacerbating in the year with irregular rainfall (from June to September). Among factors such as soil texture, soil fertility, and orchard management, the soil sand proportion, soil calcium, soil potassium, and soil magnesium content were the main factors contributing to the occurrence of fruit cracking, with contributions of 18.57%, 17.14%, 10.00%, and 8.75%, respectively. Fruit cracking was significantly positively correlated with soil magnesium content (0.802) and significantly negatively correlated with soil calcium (0.8007), potassium (0.7616), and soil sand proportion (0.7826). The integrated management treatment (organic fertilizer to improve soil + foliar nutrient supplementation) showed better control on fruit cracking by 9.34–65.25% and an increase in yield by 4.13–37.49%, respectively, compared to the supplementation of a single element in all orchards with different production and quality traits. Our findings could thus help citrus growers optimize cultivation techniques for quality citrus production under increasingly changing climatic conditions.

## 1. Introduction

Citrus is one of the most popular fruit crops, boasting the largest planting area and yield among fruit trees worldwide. Currently, citrus is produced in 135 countries globally, mainly in temperate and humid regions located below 35° north latitude and above 35° south latitude. As a perennial woody plant, citrus needs to grow in a fixed location for 20 to 50 years once planted. Therefore, site conditions and orchard cultivation management practices are extremely crucial for the growth and development of citrus. Poor site conditions, failure to improve the soil, and imbalanced fertilizer application can easily lead to low fruit quality and significant yield reduction [1,2,3,4]. Among production challenges, fruit cracking has been one of the most important production issues affecting citrus yield in recent years [5]. Fruit cracking is a common physiological disorder prominently observed in all citrus varieties [6]. Generally, fruit cracking in citrus can be divided into three types, namely, flavedo-splitting, inner-cracking, and albedo-splitting [5]. The type of cracking varies at different stages of fruit development. For example, during the fruit expansion stage, flavedo-splitting and inner-cracking are the main types of citrus fruit cracking. This type of cracking usually starts from the oil gland layer covered by the peel or the central axis of the fruit, followed by the outer peel at the top of the fruit, progressing into the middle part of the peel, and, finally, the fruit cracks along with the pulp [5]. This condition is often influenced by drastic changes in external moisture conditions, inducing rapid water uptake and expansion in the fruit within a short period [7]. The fruit pulp grows rapidly while the peel grows slowly, causing asynchronous growth between the peel and pulp, and, finally, an increased internal fruit pressure contributes to fruit cracking [8]. However, during the fruit ripening stage, albedo-splitting (fruit creasing or pitting) is the main type of citrus fruit cracking. At the beginning of this stage, the fruit appears intact on the outer peel with no obvious cracks. However, the inner peel undergoes decomposition and distortion, leading to the sinking of the outer peel and wrinkling of the fruit [9]. This type of cracking is mostly observed near the equator of the fruit, with transverse depressions in the peel coupled with wrinkling of the fruit surface.

Drastic fluctuations in external conditions that cause changes in plant water status are the most fundamental cause of fruit splitting. The transport of water in the roots and leaves primarily relies on aquaporins [10]. Therefore, any factors that can affect the water balance within the plant and its response to stress can also impact fruit splitting, such as citrus varieties, fruit size, shape, peel thickness, peel hardness, and planting conditions. Research reports have indicated that fruit size and fruit shape index have a certain influence on fruit cracking, with larger or highly oblate fruits being more prone to cracking. In addition, peel thickness and hardness are important parameters for assessing fruit cracking. Generally, thicker peel or higher peel hardness during the ripening or fruit expansion stage can greatly reduce the occurrence of fruit cracking [11]. Insufficient or imbalanced nutrient composition of the peel can lead to metabolic and developmental abnormalities, negatively affecting fruit hardness or peel thickness. For example, elements like calcium, potassium, and boron are significantly associated with the composition of pectin substances in the peel, as well as cellulose development and degradation processes, which can affect the metabolic function of cell walls, leading to decreased fruit hardness and an increased risk of cracking [12,13,14,15]. Supplementation of calcium and boron in citrus shows a positive effect on reducing fruit cracking [12]. Furthermore, factors such as rootstock, fruit hormone metabolism, light exposure, temperature, rainfall, and soil can also induce and promote citrus fruit cracking [16,17,18]. However, during the cultivation process in orchards, there are significant differences in the ecological environment and cultivation practices between different orchards, leading to variations in the effectiveness of fruit cracking prevention. How to develop targeted preventive measures based on the specific characteristics of different orchards has been a pressing issue for orchard growers. In this study, Bingtang oranges in the Xinping, China, were selected as the research subject, and 70 orchards were studied for two aspects: i. analyzing the soil texture, nutritional levels, and orchard management practices to identify limiting factors for inducing fruit cracking under different planting conditions, and ii. conducting field prevention and control experiments for fruit cracking.

## 2. Results

### 2.1. Annual Fruit Drop and Composition of Bingtang Orange in 2022 and 2023

**Figure 1:**
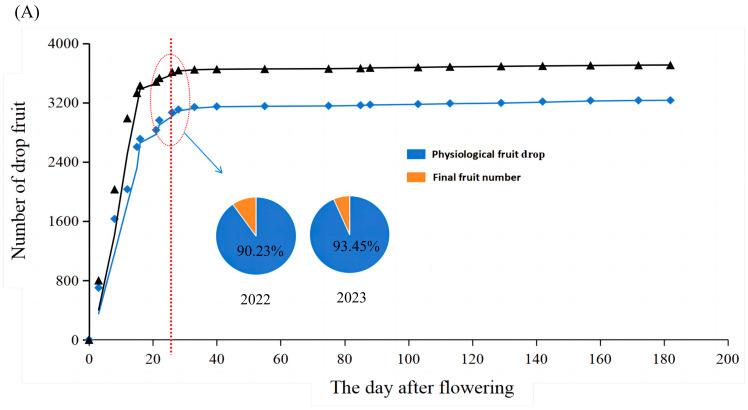

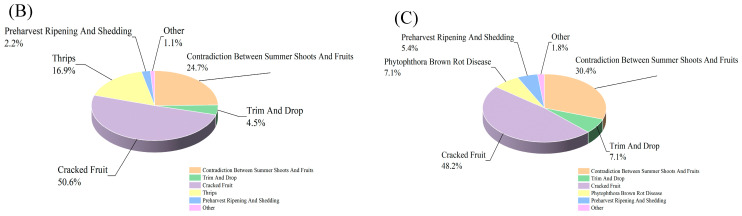
Fruit drop and related causes of Bingtang oranges in 2022 and 2023. (**A**) Fruit drop, where the blue color represents the 2022 fruit drop number, and the black line represents the 2023 fruit drop number; (**B**) the dropped fruit composition after the physiological premature drop in 2022; (**C**) the dropped fruit composition after the physiological premature drop in 2023.

This study conducted fruit drop tracking on Bingtang oranges after flowering in 2022 and 2023 (Figure 1). The number of fruits per plant in Xinping County was 3100–3900. The fruit load during the mature period was 224–330, corresponding to a yield of 40–60 kg per plant. For Bingtang oranges in the Xinping area, 0–25 days after flowering was the physiological premature drop period, and more than 90% of the fruits dropped. During the period from the end of the physiological premature drop to maturity, the occurrence of fruit drop significantly decreased by 1.4–2.5%. The fruit drop after the physiological premature drop period was mainly caused by fruit cracking (48.2–50.6%), fruit and shoot contradiction (24.7–30.4%), plant diseases and insect pests (7.1–16.9%), pruning (4.5–7.1%), and preharvest drop (2.2–5.4%), suggesting the involvement of multiple factors in fruit drop at various crop phenological stages. These observations showed that fruit cracking has become the most important factor affecting citrus yield after physiological fruit drop.

### 2.2. Rainfall Pattern in the Past 5 Years

**Figure 2:**
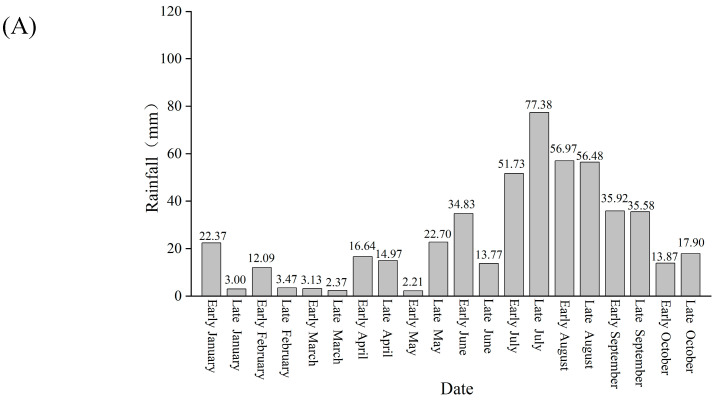

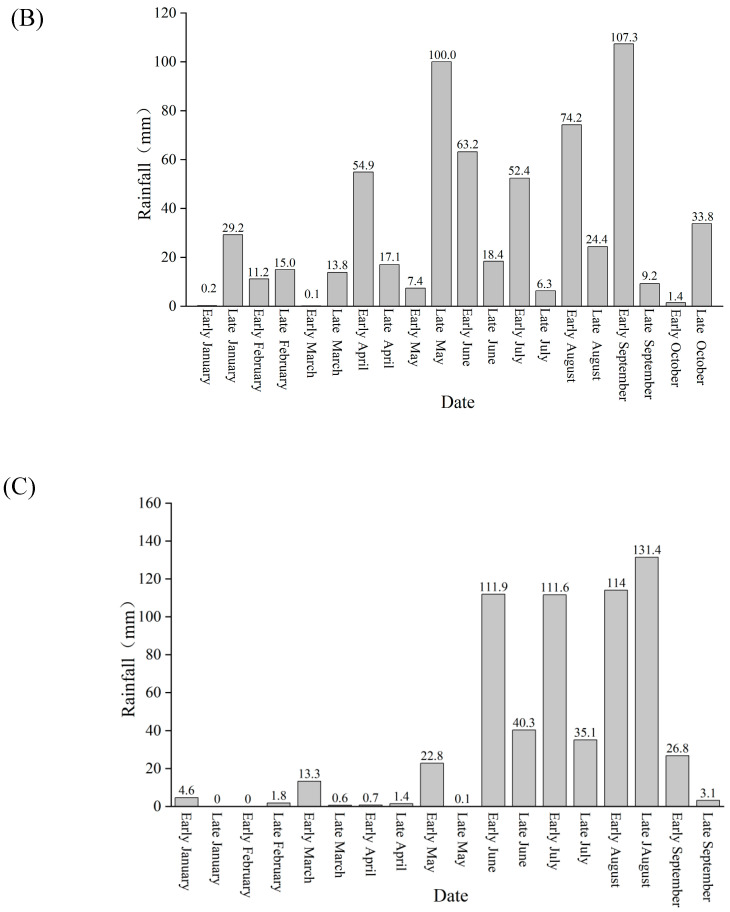
Rainfall situation in recent years. (**A**) Average rainfall from 2019–2023; (**B**) rainfall in 2022; (**C**) rainfall in 2023.

From the annual rainfall distribution in the Xinping region from 2019 to 2021, it was observed that the rainfall was relatively low from January to May, with an average monthly rainfall of only 20.59 mm. However, rainfall was concentrated from July to September, with an average monthly rainfall of 104.69 mm (Figure 2A). In comparison to 2019–2021, the rainfall in 2022 was abnormal. In 2022, the total rainfall from January to April was 141.5 mm, significantly higher than the 78.04 mm of the previous years. Additionally, the rainfall from July to September showed a pattern of higher rainfall in the first half of the month and lower rainfall in the second half, for example, 52.4 mm in the first fifteen days of July and 6.3 mm in the last fifteen days (Figure 2B). The rainfall in 2023 also exhibited significant differences, with extremely dry conditions from January to April. The total rainfall from January to April was 22.4 mm, significantly lower than the 78 mm observed from 2019 to 2021. However, there was concentrated rainfall from June to August, with a total rainfall of 544.3 mm (Figure 2C). Overall, the abnormal rainfall in the research area in the past two years created favorable conditions for fruit cracking.

### 2.3. Analysis of Cracked Fruits

**Figure 3:**
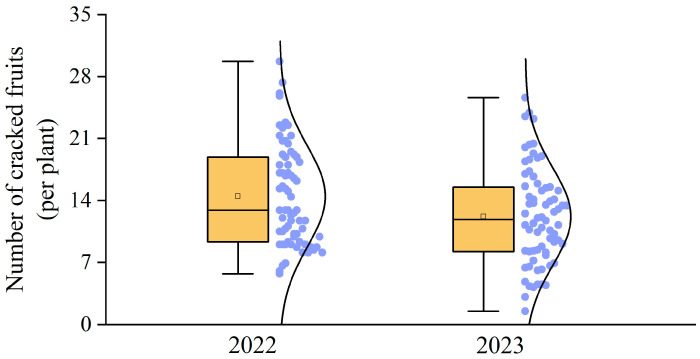
Number of cracked fruits in orchards in 2022 and 2023.

The occurrence of fruit cracking in Xinping County in 2022 and 2023 was similar (Figure 3), with the numbers being from 6 to 30 and from 3 to 25, respectively, and the average numbers were 14.03 and 13.15, respectively. The number of cracked fruits in the two years accounted for 5% to 10% of the total fruits in the respective years.

### 2.4. Soil Fertility, Physical Properties, and Orchard Management in Bingtang Orange Orchards

In 2022, 70 orchards in Xinping County were investigated for soil fertility, soil physical characteristics, orchard management measures, and fruit cracking occurrence (Table 1). From the perspective of soil fertility, the soil pH, organic matter, available nitrogen (avail-N), phosphorus (avail-P), potassium (avail-K), calcium (avail-Ca), magnesium (avail-Mg), iron (avail-Fe), manganese (avail-Mn), copper (avail-Cu), and zinc (avail-Zn) of topsoil were in the ranges of 5.34–8.10, 10.90–50.70 g/kg, 47.60–172.55 mg/kg, 22.87–236.98 mg/kg, 127.69–738.15 mg/kg, 1887.30–10,200.00 mg/kg, 70.53–601.26 mg/kg, 18.13–54.65 mg/kg, 9.17–59.22 mg/kg, 0.96–7.81 mg/kg, and 0.58–9.49 mg/kg, respectively, with the average values being 7.13, 28.22 g/kg, 94.86 mg/kg, 110.09 mg/kg, 336.27 mg/kg, 5963.26 mg/kg, 297.52 mg/kg, 34.19 mg/kg, 27.83 mg/kg, 3.32 mg/kg, and 4.87 mg/kg, respectively. Among them, the contents of exchange calcium, exchange magnesium, available copper, and available manganese in orchard soil varied greatly, and the coefficient of variation exceeded 45%. From the perspective of orchard management, the soil sand rate, soil hardness, orchard altitude, fruit hanging number per fruit tree, and fruit trees per orchard were 0.96–28.14%, 355.02–481.00 Pa, 577.99–981.53 m, 80.31–493.75, and 1942.74–3734.00, respectively. Among them, the soil sand and gravel content of orchards varied greatly, reaching 62.37%.

### 2.5. Boundary Line Analysis Between Fruit Cracking Fruit and Soil Physicochemical Characteristics and Orchard Management

**Figure 4:**
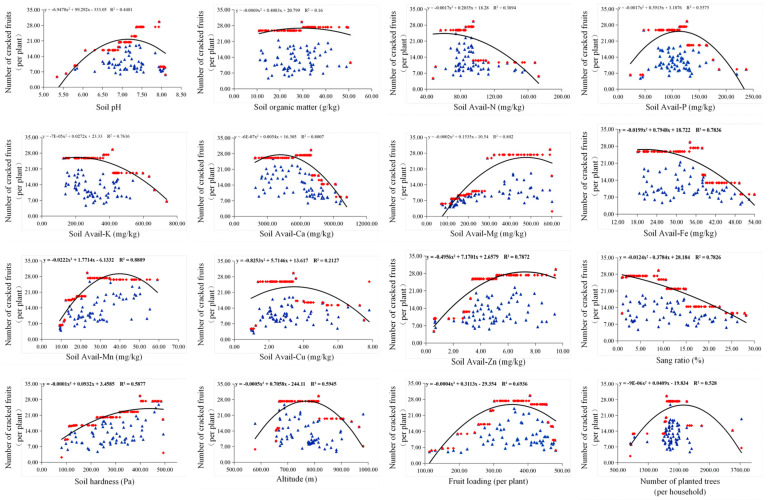
The boundary line analysis between different soil trait factors and fruit cracking. The blue triangles represent the number of fruit cracks corresponding to different factors. The red dots indicate the maximum number of fruit cracks that can be generated under different factors.

Separate boundary lines were constructed for each factor. For most factors related to cracking fruit, such as soil pH, organic matter, available nitrogen (avail-N), phosphorus (avail-P), potassium (avail-K), calcium (avail-Ca), magnesium (avail-Mg), iron (avail-Fe), manganese (avail-Mn), copper (avail-Cu), and zinc (avail-Zn), the boundary lines were polynomial curves (Figure 4). Particularly, factors like avail-K, avail-Ca, avail-Mg, avail-Fe, avail-Mn, avail-Zn, and soil sand content had a significant effect on fitting the curve with the boundary line of fruit cracking, with curve determination coefficients (R^2^) of 0.7616, 0.8007, 0.802, 0.7836, 0.8809, 0.7872, and 0.7826, respectively. An increase in soil potassium, calcium, iron, and sand-to-stone ratio tended to reduce the occurrence of fruit cracking in orchards. Conversely, an increase in soil magnesium, zinc, and manganese content could lead to more severe fruit cracking in orchards.

### 2.6. Contributions of Different Soil Trait Factors to Fruit Cracking Intensity

**Figure 5:**
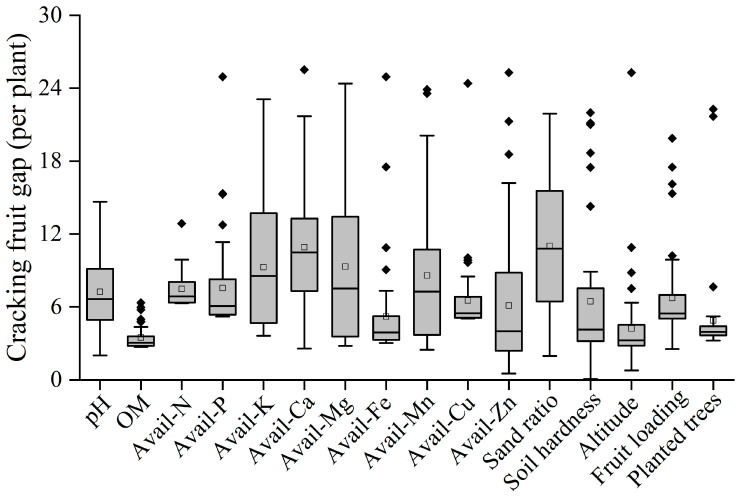
The explained cracking fruit gap for limiting factors, expressed as a percentage of the attained maximum cracking fruit in Bingtang orange orchards. The box boundaries indicate upper and lower quartiles, the whisker caps indicate 90th and 10th percentiles, and the points indicate the outliers.

The occurrence of fruit cracking under different factors could be intuitively depicted through the intensity of the fruit cracking gap. A large fruit cracking gap indicated that the factor could enhance the occurrence of fruit cracking, whereas a small fruit cracking gap signified no effect on the occurrence of fruit cracking. The 16 orchard factors obtained from boundary line fitting could explain the difference in the number of cracked fruits per plant. The soil sand rate, available calcium (avail-Ca), potassium (avail-K), magnesium (avail-Mg), and manganese (avail-Mn) showed relatively higher fruit cracking gap values of 11.02, 10.92, 9.26, 9.31, and 8.59, respectively (Figure 5), indicating that these five evaluated factors were the major contributing factors to the difference in the amount of fruit cracking per plant.

### 2.7. Orchard Obstacles Causing Fruit Cracking in Bingtang Orange Orchards

The fruit cracking-limiting factors identified through boundary line analysis and their corresponding contributions (proportions) are as follows. The largest fruit cracking gap was accounted for by the sand ratio (18.57%), followed by soil-available calcium (17.14%), potassium (10.00%), magnesium (8.75%), and manganese (7.14%). By analyzing the boundary line, fruit cracking difference, and contribution rate, it is demonstrated that soil sand content, exchangeable calcium, available potassium, exchangeable magnesium, and available manganese content are the main factors influencing the number of citrus fruits cracking.

### 2.8. Effects of Different Preventive Treatments on Fruit Cracking

Among the six citrus orchards, the number of cracked fruits per plant in the soil-hardened orchard showed an increasing trend compared to other orchards. This finding is consistent with the results of the boundary line analysis. Compared to the control treatment, all treatments except for +K showed a decreasing trend in the incidence of cracked fruits. The +DD+OM+Ca+K treatment significantly reduced the number of cracked fruit trees per plant compared to other treatments. Meanwhile, the performance of other treatments varied in orchards with different characteristics. In high-magnesium orchards, adding additional potassium supplementation not only failed to reduce fruit cracking but also tended to increase its occurrence (by 1.51% and 7.64%). However, increasing calcium and potassium while reducing magnesium showed the highest prevention efficiency for fruit cracking (46.18%). In low-potassium orchards, increasing potassium while also increasing calcium significantly decreased the number of fruit cracking (by 58.95%), compared to only adding potassium treatment (11.18%). In low-calcium orchards, supplementing calcium could significantly reduce the occurrence of fruit cracking (by 26.77% and 42.36%), but supplementing calcium while increasing potassium and reducing magnesium showed better preventive efficiency (by 30.64% and 49.50%). For the orchard with hardened soil, cultivation measures such as adding organic fertilizer and dredging drainage ditches could significantly reduce fruit cracking in the orchard. Additionally, supplementing calcium and potassium elements significantly decreased the fruit cracking number by 50.90% compared to the treatment of adding organic fertilizer and by 46.03% compared to the treatment of dredging drainage ditches.

### 2.9. Effects of Different Treatments on the Yield of Bingtang Orange

The application of nutrients and organic fertilizers had a significant impact on the yield of high-magnesium, low-potassium, low-calcium, and soil-hardened orchards. Among the six citrus orchards, the yield per plant in the soil-hardened orchard significantly decreased compared to the other orchards. There was a significant relationship between the yield of orchards and the effectiveness of different treatments in controlling fruit cracking, and controlling fruit cracking could significantly increase the yield of orchards. The use of organic fertilizers increased the yield of orchards with various traits. In high-magnesium orchards, increasing calcium and potassium elements while reducing magnesium application could significantly increase orchard yield. In low-potassium orchards, the addition of organic fertilizer and calcium significantly increased the yield by 8.03% and 17.50%, respectively. In low-calcium orchards, the addition of organic matter treatment, calcium supplementation treatment, +Ca+K-Mg treatment, and +DD+OM+Ca+K treatment significantly increased the orchard yield by 14.19%, 10.38%, 15.20%, and 24.49%, respectively. In soil-compacted orchards, using organic fertilizers and dredging drainage ditches promoted an increase in orchard yield. In summary, there is a close relationship between the yield of orchards and the occurrence of fruit cracking. Adjusting the nutrition of calcium, potassium, and magnesium in orchards, as well as using organic fertilizers and dredging drainage ditches, increased the yield of citrus orchards with a severe amount of fruit cracking. However, the measures taken for orchards with different soil conditions should be adjusted accordingly; otherwise, the yield improvement effect will be significantly reduced.

## 3. Materials and Method

### 3.1. Orchard Sites and Meteorological Data

The study was conducted in 70 Bingtang orange (*Citrus sinensis* L. Osbeck cv. *Bingtang*) orchards managed by Xinping Chushi Agriculture Co., Ltd., China (101°32′ E, 24°6′ N). The planting density of these orchards is 1000–1500 plants per hectare with an inter-plant distance of 3 m. Each orchard has an area of 3–4 hectares and follows the same cultural procedures, including fertilization, pesticide application, and plant management (pruning, etc.). As many as 15 uniform and vigorously growing 11-year-old plants were selected for sampling and counting fruit cracking in an “S” shape, and they were marked with a red sign.

The meteorological data from 2019 to 2023 were obtained from two meteorological stations (RT001, TianQi, Yunnan, China), which were evenly distributed in the orchards. The meteorological stations collected data on the area hourly and uploaded it to an online database (https://ecois.info). Hourly rainfall data were retrieved from the online database (https://ecois.info) and processed by using Excel.

### 3.2. Soil Sample Collection and Determination

The soil samples from 70 Bingtang oranges were collected in November 2022. Two soil holes, 1 m–1.2 m away from the trunk and evenly distributed under the tree canopy, were made to collect soil samples. The soil samples at 0–30 cm depth of 15 trees were collected and mixed to develop one composite sample. Then, we retained approximately 1.5 kg of a soil sample by using the quartering method for nutrition determination and soil sand proportion. All soil samples were placed in a cool and ventilated room to dry and then crushed with a wooden roller. During the crushing process, all stones of the soil samples were carefully picked out and then weighed to calculate the proportion of sand in the soil using the following formula: the sand proportion = the sand weight/the soil weight. The soil pH was measured by a pH meter (PHS-3C, Lei-Ci, China). The soil organic matter was treated with 0.8 mol L^−1^ K_2_Cr_2_O_7_ and determined by o-Phenanthroline titration. The alkaline hydrolysis diffusion method was used to determine the soil-available N. Available P was determined by Olsen’s method. Available K was extracted with 1 mol L^−1^ NH_4_OAC and determined by a flame photometer (FP6410, INESA, Shanghai, China). Available Ca and Mg were extracted with 1 mol L^−1^ NH_4_OAC and determined by an AAS (NovAA 800F, Analytik Jena, Germany). Soil-available Fe, Mn, Cu, and Zn were extracted with DTPA (0.005 mol L^−1^ TEA, 0.01 mol L^−1^ CaCl_2_, pH 7.3 ± 0.05) and determined by an AAS (NovAA 800F, Analytik Jena, Germany). Detailed measurement processes referenced the studies of Zhang et al. [19] and Dong et al. [3].

### 3.3. Fruit Drop and Cracked Fruit

The fruit drop was measured from February 2022 to November 2023. A total of 2 orchards and three trees per orchard out of 70 orchards were selected to conduct fruit drop statistics work. A plastic windproof net with pores less than 4 mm was laid under the tree canopy and covered the entire area of every plant. During the physiological premature drop of citrus, the fallen fruits on the net were counted and removed from the net every 2–4 d. From the enlargement stage to harvest, the fallen fruits on the net were counted and removed from the net every 10–16 d. The counting of the number of cracking fruits was undertaken from August to November of 2022 and 2023. The cracking fruits of 15 marked citrus plants of 70 orchards were counted and removed every 15 d.

### 3.4. Experimental Design of Fruit Cracking Prevention

The experiment of fruit cracking prevention was carried out in 4 Bingtang orange (*Citrus sinensis* L. Osbeck cv. Bingtang) orchards in Yuxi, Yunnan, China (101°32′ E, 24°6′ N), and in 2 Citrus unshiu Marc. (Satsuma Mandarin) orchards in Taizhou, Zhejiang, China (121°15′ E, 28°36′ N). The physicochemical properties of quaternary red soil (0–30 cm) of four orchards were as Table 2 shows. Based on the nutrition of these six orchards, 8 treatments were designed as follows: CK (control, farmer management pattern); +OM (farmer management pattern + 2 kg organic fertilizer); +DD (farmer management pattern + Drainage ditch); +Ca (farmer management pattern + 0.2%Ca, foliar spraying three times in May, June, and July); +K (farmer management pattern + 0.2%K_2_SO_4_, foliar spraying three times in May, June, and July); +Ca+K (farmer management pattern + 0.2%K_2_SO_4+_0.2%Ca, foliar spraying three times in May, June, and July); +Ca+K-Mg (farmer management pattern (remove the three times of magnesium fertilizer sprayed on the leaf + 0.2%K_2_SO_4_ + 0.2%Ca, foliar spraying three times in May, June, and July); and +DD+OM+Ca+K (farmer management pattern + 0.2%K_2_SO_4_ + 0.2%Ca foliar spraying three times in May, June, and July + 2 kg organic fertilizer + Drainage ditch). The effects of different treatments on the cracked fruit occurrence and yield were studied with four replicates and 5–7 citrus plants for each replicate by a randomized block design. The cracked fruits under all treatments were counted from August to November by the method described above. The yield of different treatments was measured in November by weighting all of the fruits harvested.

### 3.5. Cracked Fruit-Related Factors and Factor Contribution Analysis by Boundary Line

The boundary line analysis was used to study the comprehensive impacts of production factors, including soil sand proportion, soil hardness, soil nutrient status, tree loading, etc., on the occurrence of Bingtang orange fruit cracking. The boundary line analysis was proposed by Webb and reflected the optimal trend performance of the dependent variable at different levels of independent variables [20]. This method was reported widely in quantifying crop yield limitations and evaluating crop yield improvement potential under large sample conditions [21,22,23]. The steps involved in boundary line analysis consisted of (i) conducting the scatter plots for a range of influence factors and cracked fruit numbers and (ii) identifying the upper boundary points, built from scatter plots with the boundary line development system (BOLIDES). The description can be simplified as follows: the independent variables X (soil hardness, soil gravel percentage, soil pH, soil nutrient content, etc.) were paired with the dependent variable Y (number of cracked fruits in the orchard) one by one by using an IF statement: sort the independent variables in ascending order and the dependent variable in descending order. We applied the IF(Yi + 1 > Yi, Yi + 1, Yi), where i = 1, 2, 3, 4… to sort out the sequence after the maximum boundary value. Finally, the determination of the maximum boundary value was completed, and we performed (iii) fitting of the curve to the upper boundary points by the binary regression line.

### 3.6. Statistical Analysis

Unless otherwise noted, the results are given as means ± standard error (SE). Data were analyzed using analysis of variance (ANOVA), and the differences between means were determined by the least significant difference (Duncan) test at *p* < 0.05. The SPSS PASW Statistics 18.0 analytical software package, origin Pro 2018, and R studio were used for all statistical analyses and picture production.

## 4. Discussion

During the growth and development of citrus fruits, fruit splitting is a common physiological phenomenon that typically occurs between July and November. In the fruit expansion stage, splitting mainly manifests as yellow tissue splitting, usually starting from the bottom of the fruit, with the crack gradually widening, ultimately leading to the fruit rupturing [24]. The causes of fruit splitting are diverse, including citrus varieties, cultivation methods, adverse growing conditions, irregular rainfall, and imbalanced plant nutrition. Research indicates that enhancing nutrition, such as supplementing with calcium, boron, potassium, and applying plant growth regulators like GA3 and 2,4-D through foliar spraying, may effectively prevent fruit splitting [11,13,14,25]. Gibberellin (GA3) is a key substance that regulates fruit splitting by increasing the plasticity of the cell wall, but its application may also delay the breakdown of chlorophyll, affecting the color transformation of the fruit, leading to inconsistent evaluations of the effectiveness of growth regulators [26]. Additionally, the deficiency of mineral elements such as phosphorus, calcium, and potassium can also affect the thickness of the fruit peel, thereby influencing the occurrence of fruit splitting. Similar to previous research, our study indicated that Ca, Mg, and K were the three dominant elements in mineral nutrition associated with fruit cracking. Boundary line analysis results suggested that the occurrence of cracked fruit in Bingtang orange orchards significantly decreased with increases in soil-available calcium (R^2^ = 0.8007) and potassium (R^2^ = 0.7616) contents, while significantly increasing with increasing soil-available magnesium (R^2^ = 0.8020) (Figure 4). Different applications of potassium fertilizer significantly decreased the occurrence of cracked fruit in Ehime Kashi No.34, a high-incidence cultivated variety, by influencing the physiological metabolism process of citrus fruit peel and effectively increasing the nutrient content of calcium, nitrogen, and other nutrients in the peel [15]. Calcium is widely involved in the fruit development process and cell wall metabolism of citrus fruits. The application of calcium significantly affects the composition of pectin in the cell wall of citrus fruit peel, increasing the proportion of Ca-bound pectin and decreasing pectin decomposition [27,28]. The calcium content in the peel of cracked fruits was reported to be significantly lower than that in fruit peels with an optimum calcium level. Increasing calcium nutrition levels is currently the most widely used technique in citrus production [27]. In addition to soil nutritional factors, the occurrence of cracked fruit in Bingtang orange is also significantly influenced by soil physical properties, such as soil sand properties (R^2^ = 0.7826) and soil hardness (R^2^ = 0.5877). These variations in soil physical properties adversely affect the root system architecture, resulting in lower dry matter, total root length, and surface area in compacted soil [29,30]. Throughout the fruit development period, poor soil characteristics cause a series of difficulties in soil drainage and aeration under abnormal climate conditions, inducing the occurrence of cracked fruit [31].

**Figure 6:**
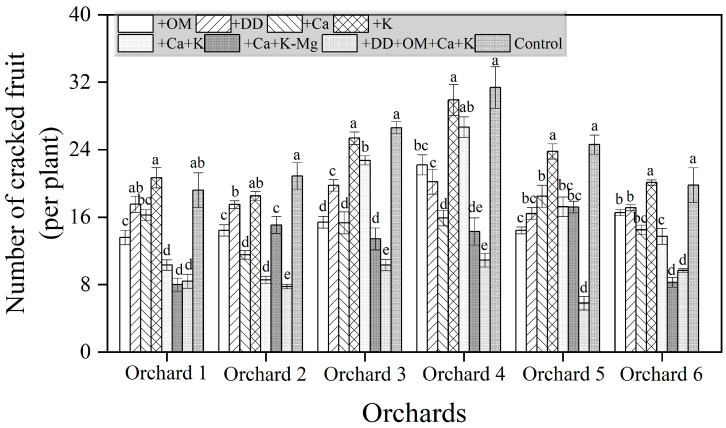
The effects of different treatments on controlling citrus cracking. Different lowercase letters indicate significant differences among different treatments at 5% level (*p* ≤ 0.05).

**Figure 7:**
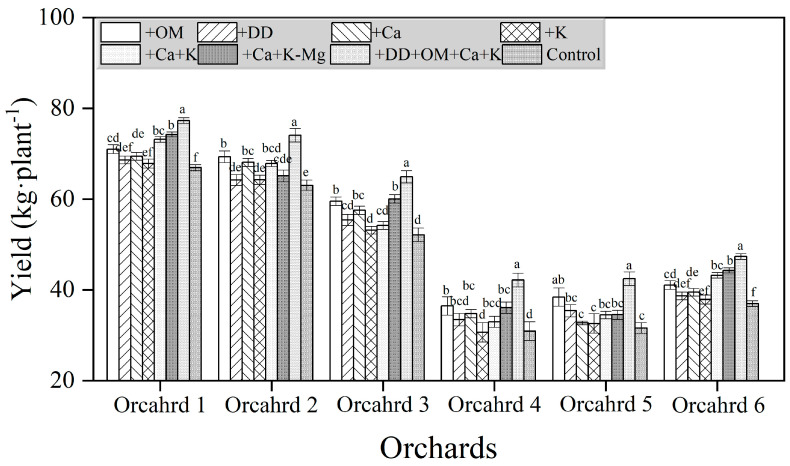
The effects of different treatments on the yield of different citrus orchards. Different lowercase letters indicate significant differences among different treatments at 5% level (*p* ≤ 0.05).

**Figure 8:**
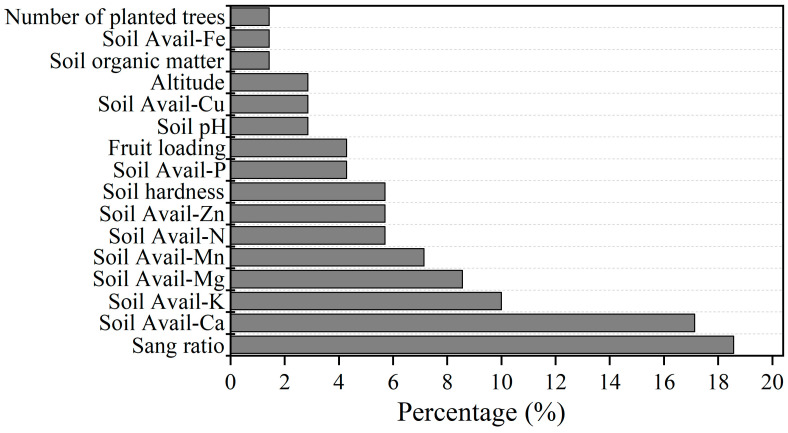
Different soil trait factors identified from the boundary line analysis and their corresponding contribution (proportions) for citrus cracking.

In this study, the results of eight treatments indicated that the +DD+OM+K+Ca treatment demonstrated the most effective prevention against cracked fruits in different types of orchards (orchards with potassium deficiency, calcium deficiency, magnesium excess, and high soil hardness), regardless of whether it is oranges or mandarins (Figure 6). As a perennial woody plant, citrus remains rooted in one stationary site for decades. The multiple cycles of root and shoot growth each year make citrus cultivation highly complex, involving both rhizosphere biology and the physiology of the above-ground canopy, including the relationship between the below-ground and above-ground plant portions. Therefore, comprehensive soil-tree management appears more conducive to addressing industry challenges like fruit cracking than applying a single element during citrus production (Figure 6 and Figure 7). The corresponding contributions (proportions) of different soil trait factors identified from boundary line analysis for citrus cracking showed that the largest fruit cracking gap was accounted for by the sand ratio (18.57%), followed by soil-available calcium (17.14%), soil-available potassium (10.00%), soil-available magnesium (8.75%), and soil-available manganese (7.14%) in decreasing order (Figure 8). Based on the comprehensive analysis of orchard characteristics, the organic fertilizer increase treatment, designed to address issues such as high soil hardness and compaction, showed positive effects on fruit cracking prevention and control (Figure 6). The application of organic fertilizer by ditching can improve soil properties, promote deep root penetration, and reduce the impact of external environmental fluctuations on the nutrient and water absorption of citrus [32]. Applying organic fertilizer effectively promotes the growth and development of citrus roots, enhancing the plant’s resistance to stress [33]. These changes collectively have a positive effect on preventing fruit cracking. Frequent occurrences of extreme climatic conditions, such as alternating heavy rainfall and drought, are highly likely to impact the functionality of plant plasma membrane intrinsic proteins (PIPs) [34]. Specifically, alterations in PIP function may lead to an increased rate of water uptake by citrus fruit cells, subsequently causing cell expansion [35]. These changes can, to a certain extent, facilitate the occurrence of fruit cracking. Moreover, the nutritional status of plants, particularly the concentrations of Ca and K, as well as the high permeability of the soil, is considered to play crucial roles in the regulation of PIPs [36,37]. When integrated measures are taken to modulate these factors, they hold promise as an important approach to reducing the incidence of fruit cracking in citrus.

Calcium (Ca) emerges as the second major factor influencing the occurrence of fruit cracking in Bingtang orange orchards (Figure 6). Foliar spraying of Ca nutrition positively prevents fruit cracking in orchards with low Ca, low K, and high Mg [38]. However, its efficacy is less prominent in orchards with hardened soil. Similar results are observed in treatments involving single K nutrition supplements compared to combined Ca and K supplements. Notably, significant variations are witnessed in the preventive effects of fruit cracking across orchards with different characteristics. Measures that fail to consider the specific circumstances of orchards yield no preventive effects and lead to orchard nutrition imbalances, negatively impacting citrus production [39]. Therefore, a targeted and comprehensive orchard management plan proves more effective in addressing production challenges than single-factor management. In this study, we adopted a systematic management approach to address the issue of fruit cracking in orchards, which involves the following steps: (i) conducting investigations and surveys to understand the production situation in specific citrus-producing areas; (ii) utilizing analytical methods to elucidate the contributions of different factors to fruit cracking and sorting these contributions based on their relevance; and (iii) strengthening the weakest aspects according to the orchard’s characteristics to achieve higher input use efficiency.

## 5. Conclusions

This study tackles the intricate challenge of applying the findings from single-factor studies under controlled conditions to the complexities of actual citrus production, particularly in the context of climate change. Focusing on the control of fruit cracking in Bingtang orange orchards, our research offers valuable insights into developing an integrated management system for preventing cracked fruits, an essential element for the future of citrus production. The study successfully clarifies the diverse contributions of various influencing factors to fruit cracking in the local area through extensive surveys covering a wide range of orchards. The crucial takeaway underscores the need for customized prevention and control measures, recognizing the specific characteristics of different orchards to enhance the overall effectiveness of fruit cracking prevention. This holistic approach provides a roadmap for future research and industry practices, emphasizing the integration of diverse factors and management strategies to optimize citrus production. As the citrus industry continues to evolve, this study contributes to the ongoing dialogue on sustainable and efficient practices, ensuring the resilience and prosperity of citrus orchards in the face of complex challenges.

## Figures and Tables

**Table 1:** Soil fertility, soil physical characteristics, and yield in Bingtang orange orchard.

Orchard Traits	Average	Maximum	Minimum	SD	CV (%)
pH	7.13	8.10	5.34	0.07	8.61
OM (g/kg)	28.22	50.70	10.90	1.12	33.19
Avail-N (mg/kg)	94.86	172.55	47.60	2.96	26.07
Avail-P (mg/kg)	110.09	236.98	22.87	4.44	33.57
Avail-K (mg/kg)	336.27	738.15	127.69	16.12	40.00
Avail-Ca (mg/kg)	5963.26	10,200.00	1887.30	259.96	36.27
Avail-Mg (mg/kg)	297.52	601.26	70.53	19.03	53.95
Avail-Fe (mg/kg)	34.19	54.65	18.13	1.15	28.18
Avail-Mn (mg/kg)	27.83	59.22	9.17	1.56	47.24
Avail-Cu (mg/kg)	3.32	7.81	0.96	0.19	48.56
Avail-Zn (mg/kg)	4.87	9.49	0.58	0.25	43.40
Sand ratio (%)	12.36	28.14	0.96	0.92	62.37
Soil hardness (pa)	1942.74	3734.00	828.00	13.55	22.57
Altitude (m)	771.17	981.53	577.99	10.05	10.92
Fruit loading	290.57	493.75	80.31	12.25	38.89
Planted trees/household	355.02	481.00	92.00	52.53	28.82

Notes: SD: standard error; CV: coefficient of variation; OM: organic matter.

**Table 2:** The physicochemical properties of quaternary red soil (0–30 cm) of four orchards.

Nutrient Contents	Bingtang Orange Orchards				Mandarin Orchards	
	Orchard 1	Orchard 2	Orchard 3	Orchard 4	Orchard 1	Orchard 2
pH	6.37	6.65	6.41	6.12	5.98	5.74
OM (g kg^−1^)	15.57	17.10	16.21	9.57	10.02	21.28
Avail-N (mg kg^−1^)	80.34	88.19	85.25	76.03	76.6	82.5
Avail-P (mg kg^−1^)	18.62	15.34	17.58	17.02	39.35	38.07
Avail-K (mg kg^−1^)	154.52	68.52	168.10	155.81	100.35	132.64
Avail-Ca (mg kg^−1^)	3185.68	4286.12	1125.58	4399.47	3846.83	2074.92
Avail-Mg (mg kg^−1^)	305.81	155.21	121.64	137.52	169.35	368.92
Hardness (Pa)	226.56	259.22	286.25	473.56	458.63	249.28

## Data Availability

The original contributions presented in this study are included in the article. Further inquiries can be directed to the corresponding author.
